# Twitter-aided decision making: a review of recent developments

**DOI:** 10.1007/s10489-022-03241-9

**Published:** 2022-02-26

**Authors:** Yihong Zhang, Masumi Shirakawa, Yuanyuan Wang, Zhi Li, Takahiro Hara

**Affiliations:** 1 Graduate School of Information Science and Technology, Osaka University, Osaka, Japan; 2 Graduate School of Sciences and Technology for Innovation, Yamaguchi University, Ube, Japan

**Keywords:** Twitter, Social media, Decision support, Methodological review

## Abstract

Twitter is one of the largest online platforms where people exchange information. In the first few years since its emergence, researchers have been exploring ways to use Twitter data in various decision making scenarios, and have shown promising results. In this review, we examine 28 newer papers published in last five years (since 2016) that continued to advance Twitter-aided decision making. The application scenarios we cover include product sales prediction, stock selection, crime prevention, epidemic tracking, and traffic monitoring. We first discuss the findings presented in these papers, that is how much decision making performance has been improved with the help of Twitter data. Then we offer a methodological analysis that considers four aspects of methods used in these papers, including problem formulation, solution, Twitter feature, and information transformation. This methodological analysis aims to enable researchers and decision makers to see the applicability of Twitter-aided methods in different application domains or platforms.

## Introduction

There are several ways to view Twitter, an internet service founded in 2007. Some consider it as a microblogging platform [28, 51, 60]. Some consider it as a social network [43, 46, 80]. Some consider it as a media consisting of user generated contents [11, 110]. Some consider it as a location-based service [17, 25]. Indeed Twitter offers a variety of ways for people to interact with the world. People can create *tweets*, short messages of a length up to 140 characters,^1^ which can be anything in their mind [101]. When posting from smart phones, tweets can be tagged with geo-coordinates to include location information [36, 109]. People can follow other people, some of whom are their friends, some are just famous people [12, 19]. People can read news posted by government or news outlet accounts, and they can *like* or *retweet* what they read [48]. Whatever it is, Twitter has been a successful service. Even in 2011, 140 million tweets were posted in one month, and 460 thousand new users joined Twitter in one day.^2^ Since then, Twitter has kept a steady pace of growth, and in 2019 it has 330 million monthly active users, who post 500 million tweets each day.^3^

One may intuitively think that anything that enters people’s conversation could leave a trace on Twitter. Indeed, as we will show soon, researches have been linking real-world phenomena, such as stock price movement, disease spread, and traffic jam, to some Twitter data. Twitter has been supporting research efforts by providing free-of-charge and easy-to-use application programming interfaces (APIs) that allows researchers to obtain Twitter data. With some restrictions, the Streaming API^4^ can be used to monitor real-time Twitter activities, while the POST API^5^ can be used to retrieve static and past data. Essentially, anyone with some programming skills can instantly retrieve a large amount of data from Twitter to use in their analytics tasks.

2010-2015 is a period of initial exploration when several important works showed the potential of using tweets in various decision making scenarios. Here are some examples. In a 2010 work, Sakaki et al. proposed to use tweets to detect earthquakes in Japan [76]. By classifying earthquake-reporting tweets and inferring earthquake movement from tweets’ geo-tags, they showed that their system was able to send alarms to users in the affected area earlier than official reports. Also in 2010, Asur and Huberman proposed to use tweets to predict the sales of movies [6]. They found that the volume of tweets mentioning keywords regarding a movie correlates positively to movie revenue. In 2010 and 2012, two works proposed using tweets to predict election results. Tumasjan et al. first proposed a content analysis approach of using tweets to predict German federal election results [92]. Then Sang and Bos proposed a sentiment analysis approach to use tweets to predict Dutch Senate election [77]. Both works found that tweets are strong indicators of election outcomes. In a 2011 work, Bollen et al. proposed to correlate tweet sentiments with stock market movement [9]. They aggregated sentiments of general tweets using a six-dimension model, and found that one particular sentiment, *calm*, had significant causal relationship with stock market movements. In a 2013 work, Culotta proposed to use tweets to help influenza monitoring [20]. Applying lightweight filtering techniques, they found that tweets could show a correlation with influenza infection and alcohol sales after data cleaning. In a 2014 work, Gerber proposed to use tweets to improve crime prediction [27]. Extending the traditional kernel-density-estimation (KDE) method, he showed that adding tweets to the model improves crime prediction accuracy in 19 crime categories out of 25.

These initial explorations showed that Twitter can be useful as a reference data source in various decision making scenarios, even though Twitter is not designed to provide such aids. We can say that researchers have invented ways to used subsets of Twitter data and information generated from them in their own problems. After initial successes, many works have followed the same direction and developed the idea further. When reviewing these later works, we focus on two research questions:

- How useful is Twitter in various decision making scenarios?
- How to use Twitter in these scenarios?

The first question is about findings. Given an established decision making problem where traditional solutions exist, we would like to know by using Twitter, how much of the results can be improved. The second question is about methodologies. We would like to know the techniques used for analyzing tweets, processing text, time series, locations, and network data, and their applicability in general. Accordingly, when writing this survey paper, we divide our discussion into two parts, first we discuss the findings, then we make a summarizing effort of methodologies and techniques involved in these works.

Several previous attempts had been made to summarize works that use Twitter in prediction problems. For example, an early attempt made by Kalampokis et al. in 2013 provided a summary of techniques used in a number of works that use Twitter and other social media in prediction problems [41]. They identified key phases of these techniques, including raw data filtering, computation of predictor, creating prediction model, and evaluation. This work was stimulating and boosted further research attempts on Twitter-aided prediction. However, since this work itself was an early work, the works it discussed did not cover newer trends. Madlberger and Almansour made a study that covered papers found by Kalampokis et al. plus some new papers in 2014 [53]. They defined five application domains, including movie, health, politics, natural disasters and crime, and finance, and selected five influential papers in each domain in the analysis. Their focus, though, was on the validity and reliability of the result, while mentioning only briefly the findings and methodologies. In a 2015 work, Prada surveyed a number of works that use Twitter in scenarios such as political election outcome prediction, crime risk prediction, disease spread surveillance, and stock market forecast, summarizing the findings in these works [67]. However, the techniques were only briefly mentioned. As the latest work in 2019, Kursuncu et al. surveyed a number of papers on Twitter-based predictive analysis, from a paradigm that separated fine-grained analysis and coarse-grained analysis [45]. They considered tweet-level prediction, such as sentiments and topics, as the fine-grained prediction, and event-level prediction, such as election outcome prediction, as the coarse-grained prediction, which was built on top of fine-grained prediction. We have found, though, in some works, such distinction of levels is unnecessary. But rather, low-level and high-level analysis are designed together to make the prediction. Moreover, some of their applications, such as demographic prediction did not have clear business implications.

The main purpose of this review is to provide an overview of recent developments in Twitter-aided decision making, which may encourage and help future researches in this topic. In summary, we make the following contribution with this review:

- Our review focuses on decision making scenarios and have clear business implication. While existing reviews generally discussed data analytics performed on Twitter, we narrow the selected works to those that have clear business impact.
- We offer in-depth examination of the methodologies. We recognize and discuss four aspects of methodologies used in this topic, including problem formulation, solution, Twitter features, and information transformation. We examine the methodology this way so that the applicability in different scenarios and platforms can be easily understood.
- We cover newer papers that came out in the last five years, in or after 2016. These papers reflect latest trends in data analytics, such as deep learning, which have not been thoroughly examined in existing reviews.

The remainder of this paper is organized as the following. In Section 2, we will discuss our paper selection criteria. In Section 3, we will discuss the findings in examined papers, trying to answer the first research question. In Section 4, we will analyze methodologies in these papers, trying to answer the second research question. Finally, we will offer some conclusion remarks in Section 5.

## Paper selection

**Fig. 1:**
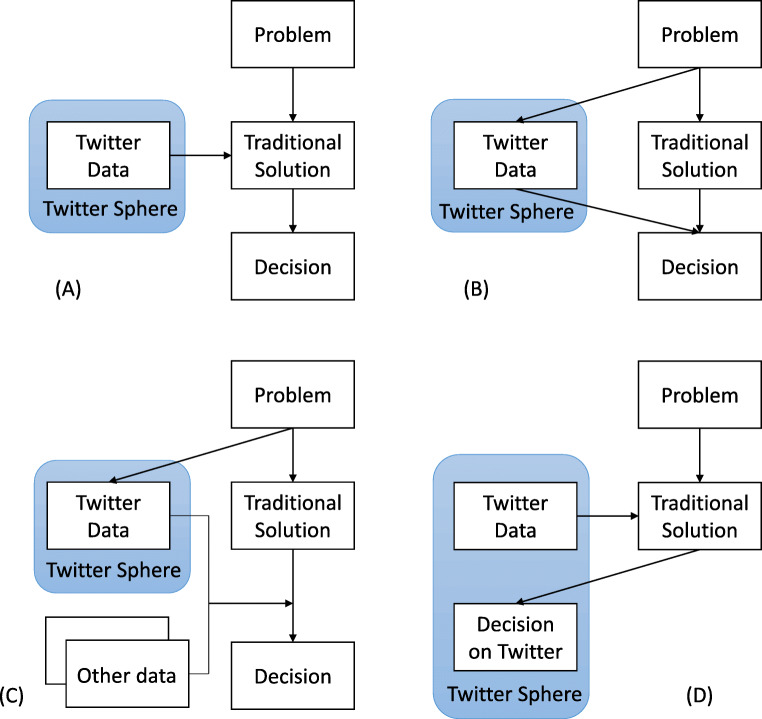
Four modes of Twitter-aided decision making

There are many kinds of data analysis that involve Twitter data. In this review, we will cover three modes that use Twitter data to help real-world decision making. They are shown as (A), (B) and (C) in Fig. 1. In all cases we require that a problem and some traditional solutions have been established. To use Twitter data, one can add Twitter data as additional information to the traditional solution (added, mode A). Or one can use Twitter data in a separate solution that can be potentially better than the traditional solution (sole data, mode B). One can also use Twitter data in combination with other data in a solution (combined data, mode C). It is worth noting here that there is a type of works that makes decision within the Twitter sphere (mode D). One example is analyzing Twitter user profiles in order to recommend products to Twitter users [26]. Even though they may still be useful for making real-world decisions, they are too platform dependent and the applicability of their solution is usually limited outside the platform. We will not cover this type of works because we focus on the side of Twitter as a helpful information source in real-world decision making, and not as the subject to pass decisions on.

Given above data usage modes, we decide on some application scenarios where Twitter-aided decision making can be performed. Previous surveys have covered scenario categories such as health, politics, natural disaster and crime, finance, stock, sales, sports events, and transportation [45, 53, 67]. From these categories, we picked five application scenarios. They are product sales prediction, stock selection, crime prevention, epidemic tracking, and traffic monitoring. We choose these scenarios because the business implication in them is clear, and for the same reason we did not include political and disaster-related scenarios. The business implication is important in this paper because it shows Twitter is not just a virtual platform, but can have acute impact on real-world operation. Even though the chosen scenarios are from diverse areas, we can see potential consequences following Twitter data analysis in all of them. Furthermore, the chosen scenarios also have attracted enough relevant research works to be reviewed comparatively.

After deciding on these scenarios, we set out to search for relevant works. Basically, we search Google Scholar^6^ using the keyword *Twitter* and *tweets*, and the name of the application scenario. Google Scholar ranks papers by relevance to the keywords and their impact, i.e., the number of citations. Therefore we only look at first 100 search results, and consider the remaining search results as either irrelevant or have little importance. After obtaining some relevant papers, we also look at the papers these papers cited, and the papers citing these papers. In this step we consider the rank of the journal or conference and citation numbers. We only pick papers from reputable journals and conferences. We recognize the reputation of journals and conference through ranking sources, including Scientific Journal Rankings^7^ and Computing Research and Education (CORE) conference and journal rankings^8^. As a result, we obtain 73 candidate papers. We then conduct first examination of the candidate paper by looking at their problem and data modes, and refine the paper using the following criteria:

- The solution proposed in the work addressed a problem that is independent to the existence of Twitter. Moreover, Twitter data was used as one of the three modes (A, B, and C) shown in Fig. 1, to help an established traditional solution. If the solution addresses a problem within Twitter sphere, like mode D in Fig. 1, it is excluded.
- The work was published on a reputable venue, and preferably have attracted relatively high number of citations, taking into account their publication year.
- The work was published in or after 2016.

Finally, we decide on 28 papers, 5 to 7 for each of the application scenarios, which are the main papers to be analyzed in this review. Although this is not an exhaustive search, we consider that the selected papers are representative for the on-going research in the field, and cover most impactful works.

## Findings regarding usefulness of twitter data

In this section, we will discuss the findings in the examined papers. The focus is on answering our first research question: how useful is Twitter in various decision making scenarios? More specifically, we discuss the improvements of solution performance, such as increases of prediction accuracy or decreases of error rates, when Twitter data is considered.

### Product sales prediction

Traditionally, predicting product sales considers many factors including competition, perceived quality of the product, state of the economy, and marketing efforts [71]. Social media, where people exchange ideas and opinions, can be an important platform for measuring product publicity [86]. In earlier years it has been shown that sales of movies and books can be predicted using online chatters [6, 32]. But such prediction requires that the products should be famous and attractive for conversations [47]. Later works have challenged this constraint.

Product sales prediction with Twitter has been generally treated as a correlation discovery problem, and the goal is to find meaningful correlation between sales numbers and relevant tweet volumes. For example, Pai and Liu proposed to use tweets and stock market values to predict vehicle sales [64]. Their target variable was the total number of vehicle sales in US and this is traditionally predicted using time series models such as ARIMA [15]. The main finding was that by using tweet sentiment score alone, the prediction of total vehicle sales number was about 2.5% better than ARIMA model, and adding stock index it was 3.2% better, measured by MAPE. Shayaa et al. made an attempt to establish the association between social media posts and the consumer confidence index (CCI), which has been shown to be a forecast signal of consumer spending [81]. Unfortunately, the statistics showed that the correlation between social media sentiment scores and the published CCI had very weak correlations, even though they claimed that sentiment analysis could replace the survey-based method for measuring CCI. Tsiara and Tjortjis made a study of predicting chart position of songs using tweets [91]. They collected tweets mentioning song names, and run correlation analysis between number of tweets and several other variables, such as chart position, song play count, and artist sentiments. They found that there is a moderate correlation of 0.292 between number of tweets mentioning and the success of the song in the following week.

In some other works, event is studied instead of sales numbers. Events are usually considered as anomalies in a time series, such as peaks that deviate largely from the mean. Some temporal activities, such as flash sales, can be also considered as events. Kolchyna et al. made an attempt to predict sales spikes using Twitter [42]. The main finding from this work was that for some event types (typically a sharp rise followed by a slow fall), tweets transformed into sentiment time series could be used to predict product sales events slightly better than random prediction. But for other event types there was no evidence that the prediction was better than random. Furthermore, the predictiveness was measured retrospectively in past data, and was not tested in new data. Zhang et al. studied the problem of predicting flash sales success with signals discovered from tweets [105]. Based on a flash sales dataset of 2,000 sales that have a binary flag indicating successful or not, their task was to find words that were predictive for sales success. Dividing the dataset into training and test periods, they showed that the discovered timing signals are useful for predicting future flash sales success. Compared to using only sales information, they showed that prediction precision could be improved by 5% to 10% after adding the signals discovered from tweets.

### Stock selection

Stock market prediction works have in many years assumed the efficient market hypothesis, which states that the current market price fully reflects all available information [68]. Although some works have proposed alternative approaches such as event detection [23, 73], the most prominent task for data-centric approach to stock prediction is to find a way to grasp relevant information [34]. Early works on predicting stock market with Twitter have shown that aspects of tweets, such as sentiments, are potentially predictive for stock market changes. For example, Bollen et al. have shown that, after analyzing seven categories of moods extracted from tweets, the mood “calm” is positively correlated to Dow Jones Industrial Average (DJIA) index 3-4 days later [9]. Recent works have pursued this direction further. We review recent paper on prediction in individual stocks instead aggregated indexes, because for investors to make investment decision, it is more useful to predict on individual stocks.

Similar to product sales prediction, stock selection using Twitter is also often treated as a correlation discovery problem. Some works have been exploring additional information on Twitter other than tweet volumes. Sul et al. made an investigation on the effect of Twitter user influence on stock price movements [89]. Their new contribution was that they divided users who tweet about the stocks into those with many followers and those of few followers. Their hypothesis was that, based on theory of Gradual Information Diffusion model, tweets from users with few followers would take longer to spread and take effect, thus could impact stock price in the farther future. Their experiment results were consistent with their hypothesis. Modeled into a linear regression formula, sentiments of tweets from few followers gave a significantly higher adjusted *R*^2^ when stock prices up to 20-days into the future were considered, much higher than that given by sentiment of tweets from many followers. Chen et al. made an attempt to classify numbers in financial tweets using various tweet text features [14]. After examining financial tweets containing numbers, they developed a taxonomy of numbers that contain 7 categories and 17 sub-categories, such as quote, forecast, and resistance. For automatic classification, eight textual features were designed, including binary values indicating presence of certain keywords. To evaluate the practical value, a trading simulation was run on 30 stocks in DJIA, and used “forecast” sub-category extracted from tweets to make prediction. Compared with analysts’ forecast extracted from Bloomberg Terminal, which achieved 2.93% average return, the proposed method achieved a higher average return of 4.86%. Ruan et al. investigated Twitter-aided stock return prediction from a trusted user perspective [74]. Different from previous works of tweet sentiment-based stock return prediction, they assigned trustworthiness scores to users instead of treating users equally. The intuition was that the more trusted users would have higher influences on the community, thus the stock price movement. They tested their approach using a stock price dataset of eight most-tweeted stocks. Comparing to treating users equally or weighting them simply by the number of followers, using the trustworthiness score achieved higher regression accuracy for all eight stocks, with lowest p-value and highest adjusted-*R*^2^. However, the number of tweets for the stock seemed to be an important factor. With the stock of another firm that ranked 9th in number of tweets, treating users equally turned out to be better. Duz Tan and Tas extended previous works by investigating tweet sentiment correlation with international stocks, including those in U.S., European and emerging markets [24]. They performed sentiment analysis and used classified tweet count in correlation analysis with mentioned stocks. They found that found that tweets were effective predictors that allows high annual returns in all three type of markets, with the best performing market being the emerging market.

Some researches also considered Twitter activities as events. Ranco et al. made an investigation on the effect of tweet sentiments on individual stocks [70]. They collected tweets containing the symbol of 30 stock companies that form the DJIA index. Then they performed sentiment extraction by classifying tweets into positive, negative, and neutral sentiments based on a number of manually labeled training examples. The results they obtained showed that the Pearson correlations and Granger causalities were weak between tweets and stock prices. However, tweets events of different sentiments were evidently significant indicators of the direction of stock price evolution in a later time. The conclusion held true for known events such as earning announcements as well as unknown event detected from abnormal values. Wei et al. investigated another possible way to use tweets to help invest in stocks [99]. This study was based on the assumption that logarithm of stock returns generally follows a normal distribution. But in case of unusual timing, there may be extreme values in stock returns that violate this assumption. And they corresponded such unusual timing to the spikes in tweet volumes about the stock. Experimenting with one year of S&P500 stocks and related tweets, they validated their hypothesis, and found that indeed stock prices within a few days before and after a tweet volume spike deviated significantly from normal distribution in Shapiro–Wilk tests. They found that implied volatility increased sharply before a Twitter volume spike and decreased quickly afterwards. They also found that right after a Twitter volume spike, options may still be overpriced, which left an investment opportunity. Based on this finding, they designed a trading strategy. With one year of simulation data, their strategy achieved 34.3% gains, while the index only increased 12.8% in the same period.

Deep learning has also appeared as a solution in this problem. Xu and Cohen followed a deep learning approach to predict stock price movements from tweets [103]. They collected tweets containing stock symbols of 88 selected stocks, and obtained embeddings for the tweets. Instead of explicit correlation analysis, they tried to find latent driven factors from tweets and historical prices using deep learning. Their deep learning model contained three components, a market information encoder (MIE), a variational movement decoder (VMD), and an attentive temporal auxiliary (ATA). The MIE turned tweets and historical prices into embeddings. Tweets of different significance were assigned different weights through the attention mechanism. The VMD was to recurrently infer and decode the latent driven factor. The ATA at the end assigned information score and dependency score of the output of VMD. In the experiments, they tested different variations of their model, some with only historical prices, some with only tweets, some combined the two information but without the ATA component. As the result, for the task of binary stock price movement prediction, their model achieved 58% accuracy, 0.6% higher than the best baseline method, which followed a similar approach but used news articles instead of tweet as the reference information source [37]. Schnaubelt et al. proposed another systematic machine learning approach for stock price movement prediction [79]. They used several dictionaries to count the words that contain sentiments, significance, and directional information, and consider them as features. Then they tested some machine learning models including random forest, neural network, and k-nearest neighborhoods. They found that random forest was the most effective method, and could provide 6.4% annual return after taking into account the transaction cost, while the general market return was 3.3%.

### Crime prevention

A recent development in the mission to fight crime is so-called predictive policing [57]. Studies have shown that many types of crimes have temporal spatial correlation, from which forecasting models can be developed [18]. For example, one theory called near-repeat has claimed that, for street crimes such as burglary, a location where an incidence has happened will likely to have another happen in the future [82]. Traditionally, solutions for this task use models that rely on temporal or spatial continuity, such as the ARIMA [15] or kernel density estimation (KDE) [3]. Twitter data is a suitable reference source to enhance this prediction because Twitter communications and crimes both concentrate in area with dense population and both invoke spatio-temporal information. Early works have shown that tweets can be used to improve crime rate prediction accuracy, although the tweets are limited to specific news accounts [96]. Following the first paper, Gerber showed that geo-tagged tweets, not specifically filtered, could improve crime rate prediction for several crime types [27]. Since then, several works have developed the idea further.

Some works aims to extend Gerber’s model with more advanced methods. For example, Vomfell et al. added taxi flow data to the model [95]. They found that there was a significant evidence that night tweets and taxi network were correlated with property and violent crimes. Testing with a number of prediction models such as general linear model (GLM), random forest (RF), and neural network (NN), they observed that the error was largest with only traditional signals, and decreasing upon adding novel features. When using econometric models, mean squared error (MSE) dropped by almost 2 after adding new features to traditional signals. But the improvement was not very obvious when using machine learning models such as random forest. Yang et al. proposed an online crime hotspot detection system that contains a crime prediction module [104]. Dividing the city area into small regions, their problem was to detect whether a crime will present in a region or not. They added features derived from tweets and Foursquare point-of-interest (POI) to the traditional model. Evaluating using New York City crime data and several classification models such as linear regression, naive Bayes, support vector machine (SVM), NN, and RF, they found that adding tweets and POIs generally improves classification accuracy. They observed an increase of accuracy by 3.4-5.2% across different classification models. On the other hand, using tweets or POIs alone did not produce good classification results that was better than KDE. Another example that followed Gerber’s model is proposed by Ristea et al., who used the model to analyze spatial correlation after sporting events [72]. They found that after adding tweet features, such as the number of violent tweets, into the model, there is a significant improvement in the model’s predictiveness.

Some researches also proposed original models to solve the problem. Aghababaei et al. modeled crime prediction as a trend classification problem, with two classes of trends, namely increase and decrease in crime number [2]. They collected tweets without keyword filtering, thus treating all words in tweets as potential signals. Using bag-of-words to represent tweets, they trained a linear SVC classifier to link future trends with past tweets. Testing for four major U.S. cities, they found that overall, the predictability was higher for Philadelphia, Houston, Chicago compared to San Francisco. Their model showed satisfactory performance for the most of crime types, such as theft, burglary, and sex offenses with F-measure up to 0.83. However, in some type of crimes such as murder and vandalism, the approach achieved the lowest result compared to the other crimes. Zhang et al. approached crime prediction problem from a recommendation system’s perspective [107]. They assigned historical crimes into time and location units, and applied various methods to predict future crimes. They tested contextual recommendation methods such as tensor decomposition and hidden topic as factors (HFT), using tweets as the contextual information. Tweets were collected and assigned to time and spatial units, and transformed into embeddings as inputs into the model. Experimental evaluation with San Francisco crime data showed that best contextual recommendation method HFT was 5% better than KDE, in terms of AUC. Compared to recommendation methods without using tweets, such as collaborative filtering, AUC was about 10% higher. Vo et al. made an attempt to predict the crime rates in seven Indian cities through geo-tagged tweets [94]. They applied several techniques, including POS tagging and clustering, and count the frequency of crime-related tweets posted in these cities. They found that crime-related tweets are a good estimates of real crime cases, and by using tweets they can predict the ranking of crime number in seven Indian cities with 70% accuracy.

### Epidemic tracking

Traditionally, tracking epidemics such as influenza are based on clinical data or self-reporting efforts^9^. It has been shown that activities on the Web, such as search engine usages, are correlated with epidemic spread [29]. Early works used Twitter to help tracking epidemic primarily collected and analyzed tweets containing the epidemic keyword and geo-location data [83]. They also used machine learning methods to determine relevant features that are useful for predicting disease spread [75].

In some recent works, disease monitoring using Twitter has been treated as a correlation discovery problem, similar to the product sales prediction. Masri et al. studied using tweets to improve the prediction of ZIKV epidemic [55]. They collected tweets containing the keyword *zika* and *mosquito* and compared them with weekly ZIKV cases reports provided by the government. Testing several lag values, they found that tweets with one week lag reached the highest correlation with government ZIKV reports, with a 0.67 Pearson correlation coefficient, 2 to 17 percent higher than other lag values. Consequently, building an auto-regressive (AR) prediction model, they found that adding one-week-lag tweet data substantially improved future case count prediction, comparing to the model without using tweets, increasing *R*^2^ from 0.61 to 0.74. They also found that tweets with the keyword *mosquito* did not add more information to the model since they strongly correlated to tweets with the keyword *zika*. Missier et al. made an attempt to classify tweets in order to track the development of dengue epidemic [59]. The unsupervised method they proposed was based on latent Dirichlet allocation (LDA) with 2 to 8 topics. Running on a larger dataset of 100,000 tweets, they found the corresponding classes of jokes and news in the topics. However, they were unable to associate the mosquito site class, which was the only actionable message class among four classes, with a LDA topic. Despite this, they found very specific topics in LDA topics, such as the Aedes-transmitted viruses. Jahanbin et al. proposed a framework for tracking various infectious diseases, including smallpox, influenza, and malaria, based on information collected from online news and Twitter [39]. After collecting categorized news and tweets, the text went through several stages of processing, including tokenization, stop word filtering, stemming, and frequency-based filtering. Relevant terms were generated at the end of this process. A fuzzy-rule based classifier was used to classify texts to different kind of diseases, using the relevant terms. This classifier seemly outperformed other classifiers such as SVM and naive Bayes. With this framework, they monitored measles for a 183-hour period, and found that their results were consistent with government reports. Later the same classification method was also used in predicting Covid-19 outbreak [40]. An import component in such analysis is the identification of related tweets. Amin et al. proposed a solution to this problem [5]. They tested several classifiers including SVM, KNN and RF to filter positive tweets about outbreaks of dengue and flu. They found with the best classifier, RF, the precision for classification can reach 88%.

Some recent works also consider the spatial information of the epidemic, similar to crime prediction. Broniatowski et al. made an attempt to track influenza with tweets [10]. Where as previous works used Google Flu Trends to track national influenza, they combined Google Flu Trends with tweets to track municipal-level influenza. Tweets were put through three classifiers to isolate health-related, influenza-related, and case-reporting tweets. With an autoregressive integrated moving average model with exogenous covariates (ARIMAX), they found that both reference sources contained explanatory power for actual influenza case numbers, even though the model with tweets required fewer parameter than Google Flu Trends models. And since combining two sources reached a lower AIC than using individual sources, it showed that tweets contributed new predictive information that was different from Google Flu Trends. Huang et al. proposed to use Twitter data to track people’s mobility around the globe [38]. Mobility is not directed related to infections, but it is an important indicator of prevention measures. Previously based on location service data such as Google Location and Apple Maps, their method measured mobility by analyzing geo-tagged tweets from users who recorded multiple locations across time. They calculated single-day and cross-day travel distances of these users and created normalized mobility index (NMI). Their findings showed that at global level, the NMI clearly decreased after WHO declared COVID-19 as a pandemic. At country level, among studied countries, countries with the highest mobility after the outbreak were Turkey, Japan, and Malaysia, while countries with the lowest mobility were Russia, Australia, and Indonesia.

### Traffic monitoring

Traditionally, road traffic monitoring is done through sensors [7]. After the emergence of the paradigm *social sensing*, Twitter was also taken as a sensing platform [1]. It is found that social media analysis and data mining techniques are good complementary to existing methods in representing, measuring, modeling, and mining meaningful patterns in traffic events based on geosocial media data [102]. Early works have exploited the text and location information found in tweets for traffic monitoring [16]. Recent works continued to develop in this direction.

Event-based traffic monitoring in recent works still focus on tweet classification. Dabiri and Heaslip developed a Twitter-based traffic event detection model using deep learning architectures [21]. They collected a dataset of tweets and labeled them into non-traffic and traffic-related tweets. Since labeling data is an expensive and time-consuming task, they proposed an efficient labeling approach to map tweets into numerical vector space through word-embedding tools, which measured the semantic relationship between words. Experimental results on their labeled dataset showed that the proposed model achieved clear improvements over the state-of-the-art models: word-embedding models, randomly initialized word vectors, SVM, RF, and neural network classifiers. Alomari et al. proposed Iktishaf (an Arabic word meaning *discovery*, a big data tool for traffic-related event detection from Twitter data in Saudi Arabia [4]. The tool used three classifiers (logistic regression, SVM, and naive Bayes) to detect eight event types: social events, weather, fire, traffic condition, road damage, road closure, roadwork, and accident. The classifiers were evaluated using widely used numerical criteria (F-score, recall, accuracy, and precision) and classification results were validated against external newsmedia and other resources. In the evaluation, they first showed that Iktishaf Stemmer could improve tweets filtering classifiers. Then, they analyzed several traffic-related events detected from 2.5 million tweets without prior knowledge, including the KSA national day, a fire in Riyadh, rains in Makkah and Taif, and the inauguration of Al-Haramain train. Another type of approach focuses on detect traffic congestion events from tweets. Noori and Mehra proposed to use classifiers to find traffic congestion information from tweets [61]. They tested classifiers such as SVM, LR, and NB, and found that SVM was the best performing classifier, reaching 91% accuracy when classifying related tweets.

In addition to events, some researches also performed spatial analysis in traffic monitoring. Wang et al. proposed a Twitter-based railway delay detection method based on topic propagation analysis of geo-tagged tweets between railway stations [98]. They compared the performance of the proposed method with three different classification algorithms (SVM, Logistic Regression, and naive Bayes) on datasets derived from Twitter with the actual delay information from 488 stations of 62 routes in Tokyo area in Japan. Experimental results showed that the proposed method effectively detected delays and predicted their influence on railway stations, compared with the actual delay information. Gu et al. proposed a methodology to crawl, process, and filter public tweets to detect real-time traffic incident information on highways and arterial roads [33]. The core part of their procedure was the adaptive data acquisition, which establishes a dictionary with traffic-related words using a simple natural language processing (NLP) technique. Using this dictionary, they acquired a number of tweets in Pittsburgh and Philadelphia, respectively. They found that a small sample of tweets acquired covered most of the incidents reported in the existing dataset, and additional incidents could be identified through analyzing tweet text .

## An analysis of methodology in recent works

We have seen by now that Twitter data can be useful in various decision making scenarios. Now we are to analyze the methods used to achieve these results. Sometimes a method is very general and can be applied outside Twitter platform. For example, we have seen papers performed sentiment extraction on filtered tweets and related them to a real-world phenomenon such as product sales or stock price changes. This method can work as long as there are some related texts on which text sentiment analysis can be performed. However, in some papers, exclusive Twitter features such as follower and mention are taken into account, and thus cannot be easily applied in another platform. To understand the methods and their applicability, we analyze four aspects in each method, including problem formulation, solution, Twitter feature used, and information acquisition and transformation. A summary of these four aspects in each of the 28 examined paper is showed in Table 1. In this section, we will discuss in detail each of these methodological aspects.

**Table 1:** Summary of methodologies in reviewed papers

paper	application	data mode	problem	technique	feature	acquisition and transformation
[42]	product sales prediction	sole data	event detection	k-means clustering	text, time series	tweet filtering, sentiment score
[64]	product sales prediction	combined data	value prediction	SVR	text, time series	tweet filtering, sentiment score
[81]	product sales prediction	sole data	correlation analysis	Pearson’s R	text	tweet filtering, sentiment score
[105]	product sales prediction	sole data	classification	RF	text, time series	BOW
[91]	product sales prediction	sole data	correlation analysis	Pearson’s R, SVM	text	tweet filtering
[89]	stock selection	sole data	value prediction	linear regression	text, follower	tweet filtering, sentiment score
[103]	stock selection	added	classification	DNN	text	tweet filtering, word embedding
[99]	stock selection	sole data	event detection	Shapiro–Wilk test	text, time series, poster	tweet filtering
[14]	stock selection	sole data	classification	SVM, DNN	text	tweet filtering, POS tagging, word embedding
[74]	stock selection	sole data	value prediction	linear regression	text, mention	tweet filtering, sentiment score
[79]	stock selection	sole data	classification	RF, NN, KNN	text	tweet filtering, BOW
[24]	stock selection	sole data	correlation analysis	linear regression	text, time series	tweet filtering, sentiment score
[2]	crime prevention	sole data	classification	SVM	text, location	BOW
[95]	crime prevention	combined data	value prediction	linear regression, RF	location, timestamp	none (only use volume count)
[104]	crime prevention	combined data	classification	LDA, NB, SVM, RF	text, location	sentiment score, BOW
[107]	crime prevention	added	value prediction	recommendation system	text, location, timestamp	word embedding
[72]	crime prevention	added	correlation analysis	Moran’s I Index	text, location	tweet filtering
[94]	crime prevention	sole data	correlation analysis	rank comparison	text, location	tweet filtering
[55]	epidemic tracking	added	correlation analysis	AR	text, location, time series	tweet filtering
[59]	epidemic tracking	sole data	event detection	LDA	text	tweet filtering, BOW
[39]	epidemic tracking	combined data	value prediction	fuzzy classifier	text	tweet filtering, BOW
[38]	epidemic tracking	sole data	value prediction	normalized mobility index	location	none (only use volume count)
[5]	epidemic tracking	sole data	classification	SVM, KNN, RF	text	tweet filtering, BOW
[33]	traffic monitoring	sole data	event detection	LDA	text, location	tweet filtering, BOW
[21]	traffic monitoring	sole data	event detection	CNN, RNN	text, location	tweet filtering, BOW
[98]	traffic monitoring	added	classification	DNN	text, loation	tweet filtering, BOW
[4]	traffic monitoring	sole data	event detection	SVM, NB	text, location	tweet filtering, BOW
[61]	traffic monitoring	sole data	classification	SVM, LR, NB	text	BOW

### Problem formulation and solving techniques

In different application area where Twitter data is used, it is required to formulate a problem which can be solved with computational means. This problem is an abstract from the surface application. It means that different application may have a same problem formulation. For example, sales prediction and epidemic tracking can be both formulated as a correlation analysis problem [22, 55]. On the other hand, an application can be formulated into different problems. For example, in crime prevention, the problem can be value prediction [95] or classification [2]. There are usually proven computational solutions correspond to various problems, which makes solution searching easier. In this section, we will discuss the problems and corresponding techniques. Within the papers we examine, there are four problem types, namely, value prediction, classification, correlation analysis, and event detection.

#### Value prediction

One of the fundamental problems in computational intelligence is to predict a value. This value can be the stock price next day, the number of infections in an area where clinical data is absent, or the number of crimes of a neighborhood in the future. This is done usually using regression techniques in combination with a number of predictors. The regression techniques used in the examined papers include linear regression [63], random forest regression [50], support vector machine regression [84]. When the data is considered as time series, autoregression techniques (AR, ARIMAX) can also be used [90]. The solution in literature [107] was a bit unconventional. They treated crime numbers as ratings and use recommendation techniques such as collaborative filtering [78] and contextual method [35] to infer the missing number.

#### Classification

In computational intelligence settings, classification outputs a prediction that is one in many categories. If the total number of categories is two, then it is a binary classification problem. This is a very common problem especially in decision making. It gives a distinct suggestion on, for example, whether stock market will go up or down tomorrow, or whether there is a traffic jam in a specific street now. Similar to value prediction, the typical solution is to use a supervised classifier with a number of predictors. The classifiers used in the examined paper include logistic regression [52], random forest (RF) [50], support vector machine (SVM) [13], and naive Bayes (NB) [56]. These are typical classification baselines usually compared together in a paper [108]. In literature [14] unconventional deep neural networks were used to encode tweet text [44], although the framework for making classification was the same. In literature [103], a complex neural network was built for classifying stock price movement, and included many new elements in machine learning. First historical information was encoded using recurrent neural network (RNN) [87], then market information extracted from tweets was encoded using the attention mechanism [93].

#### Correlation analysis

Sometimes instead of running value prediction, the mere correlation between tweets and a target variable can reveal the usefulness of tweets. Correlation analysis focuses more on explaining the predictiveness of tweets, instead of improving prediction accuracy. Such analysis usually relies on techniques such as Pearson’s R [81] and Granger Causality [9]. Tweets are said to be predictive for the target variable, such as the number of product sales, if the lagged correlation (e.g., tweets in three days ago vs. product sales in current day) is found. Without testing on future data, though, it is hard to say if the claims are valid, and thus many works continued with more verification tests [9].

#### Event detection

While value prediction and classification try to provide a likely outcome, event detection tries to capture abnormalities. Sometimes also considered as anomaly detection, event detection has been applied to find unusual behaviors in data, such as sudden rises in sales number, stock price, or tweet frequency in a geographical area. By detecting events, one can correlate anomaly in tweets with that in, for example, stock price [99] or traffic flow [88], which cannot be easily captured with regression and classification. Long established in time series analysis [31], a typical solution is to measure quantities changes at each time step and use a threshold to output a suggestion [100]. Since tweets are used as the reference source, techniques such as k-means clustering help generate a chunk of information for processing [42]. Another common technique in finding events in tweets is latent Dirichlet allocation (LDA) [8] which can assign tweets into topics, and represent each topic by a probability distribution of words. By analyzing contents of the topics, one can grasp what actual phenomenon is happening in Twitter and how it is related to the application [110].

### Twitter feature used in data analysis

The most used Twitter feature is tweet text, which is the main body of Twitter data. Discussions of product, stock, crime, infectious disease, and traffic, are all contained in tweet text. Thus methods proposed by most examined papers involved extracting information from tweet text. In the 28 examined papers, only two papers did not consider tweet text. In literature [95], crime number is considered to be related to the volume of tweets posted in day or night. In literature [38], the volumes of tweets in different locations are considered as indicators of mobility. In such cases, tweet volume, not the text content, is used as a proxy for measuring the degree of social activities.

Apart from tweet text, other features offered by Twitter have also been used. These features include time series (built after quantifying tweets over time units), location (tagged by mobile devices or provided by the user) [17, 49], follower and following network [12], interaction indicators such as retweet or mention [74]. Time series is most useful in correlation analysis and event detection. The locations of tweets are provided either by the GPS function in the mobile device, in which case exact coordinates of the tweets are known, or by users who mention location names in the tweet text. They are used when the problem involves geographical information, for example, in geographical crime prediction and traffic monitoring. The follower and following network and interactions are mainly used to portrait certain user traits, such as authority and trustworthiness, which are used to support information found in tweet text.

### Data acquisition and information transformation

Twitter has support research community greatly by making acquisition of Twitter data easy and free-of-charge. The Filter API^10^ allows monitoring real-time Twitter postings, filtered by constrains such as user selected keywords or geo-location. The Search API^11^ allows acquiring past tweets through search keywords, although normally it will only return tweets posted within the past week.

With Twitter APIs, one can easily collect a large number of tweets. However, unstructured data such as tweet text are difficult to be used in a computational solution. Therefore most of the examined paper made efforts to filter relevant data during acquisition, and transform Twitter data into a form convenient for computational solutions.

Given a chunk of tweets, in many applications, one needs to first extract a subset of relevant tweets. This is called tweet filtering. For example, to relate tweets to stock market, it is necessary to filter tweets that discuss stocks, which usually contain stock symbols [74]. Similarly, tweets containing a brand name can be filtered when analyzing product sales [42]. Methods for filtering tweets range from keyword-based filtering to supervised machine learning. Keyword-based filtering is easy to understand, but may miss some information [62]. Supervised machine learning usually requires some feature engineering and some manually labeled training data [85]. Both methods have been widely studied.

Another popular transformation is sentiment extraction. This is an established task in natural language processing (NLP) that assigns a sentiment score to a piece of text. The sentiment score can be bi-polar (i.e,, positive and negative), or involving multiple sentiment dimensions. Similar to tweet filtering, the common methods in sentiment extraction include keyword-based method and supervised machine learning. The former makes use of a sentiment word dictionary to assign score to texts [69], while the latter involves feature engineering and manually-labeled training data [97]. Off-the-shelf software packages such as Stanford CoreNLP can perform sentiment extraction with satisfactory accuracy [54]. Once the tweet filtering and sentiment extraction were performed, one can immediately gain insights in many problems, such as the relationship between public moods and stock price [89] or product sales [81]. Because sentiment extraction in social media is effective, some works have proposed sentiment analysis as a general solution for decision making in any topic [66].

Sometimes tweet filtering is not performed because it is difficult to know beforehand which tweets are related to the problem. For example, in some Twitter-based crime analysis, all tweets are considered at first, and some words that are particularly relevant to crime can be extracted later [106]. Also in some cases when the volume of tweets is used as a proxy to measure activities, tweet filtering is also not necessary [95].

More advanced transformation techniques include part-of-speech (POS) tagging, bag-of-words (BOW), and word embedding. POS tagging is mainly used to select certain words in the text, such as nouns or adjectives [30]. BOW and word embedding are computational representations of text which are mostly used in supervised machine learning. BOW basically counts the occurrences of each word and uses them as a vector. Word embeddings are results of neural learning algorithms such as word2vec [58] or GloVe [65], and contains semantic information of each word in a vector form. Such transformations are considered necessary for more advanced analysis [103]. It is worth noting that while both have vector forms, BOW represents sparse vector while word embedding represents low-dimensional dense vector. The former can be more helpful when examining the factors in the problem based on words. The latter is more capable to fit in heavy computational models such as deep neural networks.

### Method strengths and challenges

In the above we discussed problems, solving techniques, Twitter features used, and transformation. We found that some methods have more strengths in certain setting combinations than others. For example, when solving classification problem with BOW text representation, many works used random forest classifier, which in some reports achieved best accuracy [104], comparing to other classifiers such as SVM, KNN, and naive Bayes. However, there is a limit in what random forest can achieve. In many cases, the maximum accuracy achieved in classification is around 90% [5]. To go beyond this limit may require better understanding of the data, exacting better features, if not using more suitable models.

We also see that when doing correlation analysis of tweets and a real-world phenomenon (e.g., stock price movement), the common choice is Pearson’s correlation coefficient, although linear regression is also used. However, when the number of data samples is large, the significance value in such analysis may not reflect the real correlation in the data. A special case of this problem is spurious correlation, which means a correlation was found between two variables but this is due to mere co-incident, and there is actually no relation between these two variables. The number of variables is usually large when Twitter data is considered, and avoiding spurious correlation can actually be quite challenging.

For the problem of event detection, we see two types of solving techniques. The first is supervised learning, with techniques such as SVM and naive Bayes. The second is unsupervised learning such as LDA and k-means. The advantages of unsupervised learning include removing the necessity of manual annotations, and the ability to discover unexpected new events. The disadvantage, on the other hand, is that the results from unsupervised methods are harder to interpret for human reader. We acknowledge that the unsupervised method may be a better future direction for solving event detection problem, as it has higher generality and applicability, although the interpretation issue remains a challenging problem.

## Conclusion

In this review, we analyzed 28 papers that proposed methods for using Twitter data in decision making scenarios. We first discussed the findings, which showed that decision making performances with the help of Twitter data have further improved in the past five or six years. For instances, the prediction of stock price movement has become more accurate, and the predictive policing has become more effective. Then we offered a methodology analysis, which showed whether methods proposed in these works could be applied to different problems and new platforms. And it became clear that, a set of techniques, such as tweet filtering, sentiment extraction, and correlation analysis, could be easily applied to different problems and platforms to establish links between public conversations and real-world phenomena.

However, we also see that current explorations still remain in a shallow level. For instances, the majority of works use Twitter as the sole data, with only five works out of 28 added Twitter data to existing data, and four works use Twitter data together with other data sources. There is much space to explore in multi-source data integration. With regard to techniques, only four works out of the 28 made use of the deep neural network, the latest and rapidly growing trend in computational intelligence. Moreover, only three works out of the 28 considered user and interaction features, which should contain rich information but often overlooked. New techniques such as graph mining can potentially leverage such information, but we have yet to find works that use such techniques. We can see, after analyzing the methodologies, that there is still very large space to advance Twitter-aided decision making, both in features considered, and in techniques used. We hope, by offering this review, we have made a stimulation, and provided a ground on which future researches in this topic can be built.

## Footnotes

1 In 2017, the character limit has been increased to 280.

2 https://blog.twitter.com/official/en_us/a/2011/numbers.html

3 https://financesonline.com/number-of-twitter-users/

4 https://developer.twitter.com/en/docs/twitter-api/v1/tweets/filter-realtime/overview

5 https://developer.twitter.com/en/docs/twitter-api/v1/tweets/post-and-engage/api-reference/post-statuses-update

6 https://scholar.google.com/

7 https://www.scimagojr.com/journalrank.php

8 http://portal.core.edu.au/conf-ranks/

9 Some information regarding the clinical method is provided by the US government: https://www.cdc.gov/flu/weekly/overview.htm

10 https://developer.twitter.com/en/docs/twitter-api/tweets/filtered-stream/introduction

11 https://developer.twitter.com/en/docs/twitter-api/v1/tweets/search/api-reference/get-search-tweets
